# Use of FDG PET for Staging and Re-Staging of Head and Neck Squamous Cell Carcinoma

**DOI:** 10.3390/cancers17193140

**Published:** 2025-09-27

**Authors:** Charles Marcus

**Affiliations:** 1 Division of Molecular Imaging and Therapy Service, Department of Radiology, Memorial Sloan Kettering Cancer Center, New York, NY 10065, USA; marcusc2@mskcc.org; 2 Division of Nuclear Medicine, Department of Radiology, Emory University School of Medicine, Atlanta, GA 30322, USA

**Keywords:** head and neck cancer, head and neck squamous cell carcinoma, PET/CT, PET, PET/MRI, oral cavity, oropharynx, larynx, staging, treatment response, treatment planning, follow-up, disease recurrence

## Abstract

**Simple Summary:**

^18^F-FDG PET/CT plays a complementary role to clinical evaluation in the staging, treatment planning, treatment response assessment, recurrent disease detection, and follow-up of patients with head and neck cancer. It plays both a complementary and advantageous role over conventional imaging techniques, providing an added advantage in the detection of synchronous and occult primary tumors, improving staging, and impacting treatment strategies, including surgical, radiation, and systemic treatment. It provides valuable prognostic information at different stages of diagnosis, treatment, and follow-up. The role of PET/CT in these cancers is constantly evolving, with ongoing and future trials discovering new and valuable information which can have a significant impact on the future management of these cancers.

**Abstract:**

Head and neck cancers account for approximately 3.0% of all new cancer diagnoses. 18F-FDG PET/CT plays an important role in the initial staging of these cancers, especially in the detection of nodal and distant metastatic disease, outperforming conventional imaging techniques. It helps identify occult primary tumors and synchronous second primary malignancies. PET/CT findings can lead to treatment plan alterations both in surgical and primary or adjuvant chemoradiation plans. High negative predictive value at treatment response assessment provides valuable prognostic implications. PET/CT can predict outcomes at baseline and during or after treatment.

## 1. Introduction

Head and neck squamous cell carcinomas (HNSCCs) comprise cancers of mucosal origin, arising from the different anatomic sites of the head and neck, including the oral cavity, oropharynx, nasopharynx, hypopharynx, and larynx broadly. The most affected age group is 60–70 years of age, with a higher incidence in men. The most affected site is the oral cavity and oropharynx, with the incidence rate of head and neck squamous cell cancers of the oral cavity and oropharynx (OPSCC) being approximately 18.4 per 100,000 in men and 7.5 per 100,000 in women, accounting for approximately 2.9% of all new cancer diagnoses in 2025. With accurate diagnosis and timely intervention, the 5-year relative survival is 69.5%. Laryngeal squamous cell carcinomas account for approximately 0.6% of all new cancer diagnoses, with a 5-year relative survival rate of 62.1% [1]. The most identified risk factors associated with these diagnoses are tobacco, alcohol use, and Human Papilloma Virus (HPV) infection, especially the HPV-16. There is a difference in the tumor biology, treatment response, and patient outcomes between patients with HNSCC associated with HPV and those that are HPV-negative, with tumors behaving more aggressively and less favorable outcomes. These factors also play an important role in treatment decisions with the advances in targeted therapeutic regimens [2]. Another commonly incorporated biomarker in these cancers is the tumor suppressor protein p16, which has been used as an alternative indicator of HPV infection, with p16 being high in HPV-associated tumors and HPV-negative tumors inactivating the tumor-suppressing gene encoding the p16 protein. Some strategies prefer combined evaluation of HPV and p16 status, since discrepant findings between the two can be seen in approximately 10% of these patients [3].

Imaging plays an important role in the evaluation of the extent and staging of the primary tumor, detection of locoregional and distant metastases, treatment planning, prognosis prediction, and follow-up of these patients. In recent years, there has been a significant impact of molecular imaging in the management of these patients. ^18^F-FDG PET has been shown to add important information with upstaging or downstaging disease in comparison to clinical and conventional imaging evaluation in approximately 20% of patients [4]. This article will concisely review the role of ^18^F-FDG PET in the staging and re-staging of head and neck squamous cell carcinomas.

## 2. Staging Head and Neck Squamous Cell Carcinomas

The most used clinical staging system for HNSCC staging at diagnosis is the tumor size and extent (T), regional nodal involvement (N), and distant metastasis (M), or the TNM staging proposed by the American Joint Committee on Cancer (AJCC). Imaging plays an important role in contributing information to assist in the accurate staging of these tumors [5]. Although initial imaging work-up recommendations focus on cross-sectional imaging with CT and/or MRI, ^18^F-FDG PET can be useful in certain clinical scenarios, such as detecting an occult primary, nodal evaluation in surgical and radiation treatment field planning, and in detecting distant disease, especially in patients with advanced disease, such as larger primary tumors, or in patients with nodal metastases at baseline [6]. Discussion of the staging of HNSCC in detail is beyond the scope of this article. Key details to note are that tumor and nodal metastasis characteristics, such as depth of invasion of the tumor and extra-nodal extension of nodal metastasis, which can be better evaluated with imaging modalities with superior soft tissue resolution, such as MRI, are incorporated in the staging characteristics of these tumors. With the discovery of differences in the outcomes and behavior of tumors associated with HPV in comparison to those that are not, the classification system was modified to consider these differences [7]. ^18^F-FDG PET/CT can add value in the initial staging of these tumors by changing the T, N, or M stages in almost half the patients, with the biggest impact being in the detection of additional nodal metastasis and changing baseline management decisions in almost 20% of patients [8,9]. ^18^F-FDG PET provides superior sensitivity and accuracy over conventional cross-sectional imaging techniques, such as CT or MRI, in these tumors [10].

## 3. Primary Tumor Staging

**Figure 1:**
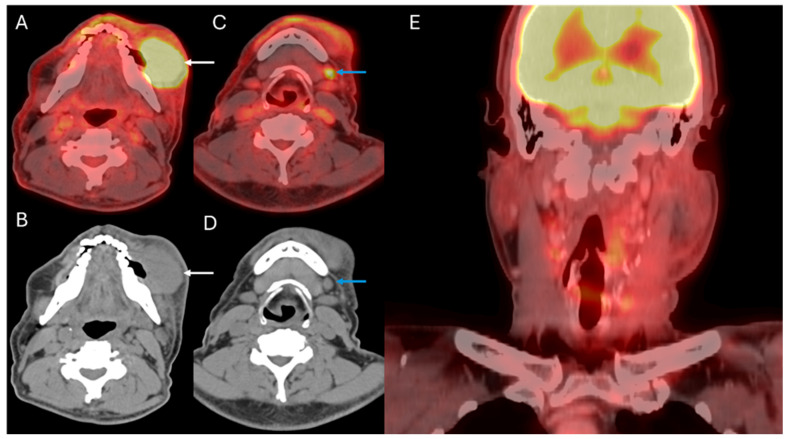
Staging and treatment response evaluation: 62-year-old male with T4aN2b oral cavity squamous cell carcinoma. Axial fused PET/CT (**A**,**C**) and axial CT (**B**,**D**) of the staging 18F-FDG PET/CT demonstrate the primary tumor involving the left buccal mucosa (white arrows) and left submandibular nodal metastases (blue arrows). Coronal fused PET/CT (**E**) of the restaging PET/CT after induction chemotherapy, buccal excision, and left neck dissection: adjuvant radiation therapy demonstrates post-treatment changes without suspicious recurrent or residual disease.

^18^F-FDG PET can detect the primary tumor in a majority of patients with a clinically detected primary tumor, limited by the size and depth of tumor invasion [11]. For primarily T stage delineation, ^18^F-FDG PET may not be the modality of choice, with CT and MRI offering adequate delineation of the margins and extent of the primary tumor, which are important for surgical and local treatment decisions, especially with the superior soft tissue resolution of MRI. However, ^18^F-FDG PET can make a significant difference in the primary tumor evaluation in patients with dental artifacts, often masking the tumor extent on conventional imaging techniques. Overall, the detection rates for ^18^F-FDG PET and MRI appear to be comparable, with some advantages of one modality over the other, as described above [10]. A multimodality imaging approach is often needed for accurate primary tumor characterization in most patients. For example, the combination of ^18^F-FDG PET/CT and contrast-enhanced CT (ceCT) provided better diagnostic accuracy than either modality alone (91% vs. 85% and 87% for ceCT and PET/CT) in identifying osseous involvement in patients with oral cavity cancers, although, in certain studies, the performance of PET/CT was comparable to ceCT with thin sections [12,13] (Figure 1).

## 4. Staging of Cervical Nodal Metastases

In the evaluation of patients with HNSCC, the detection of cervical nodal metastases, which are often not clinically apparent, can have an impact on management. Imaging plays an important role in the N classification of HNSCC, with changes in N stage on CT/MR ranging from 20% to 41% and for PET ranging from 30% to 45% [14]. ^18^F-FDG PET/CT can reduce the chance of cervical nodal metastases going undetected in clinically N0 necks, thereby significantly affecting the chances of progressive or recurrent disease with higher sensitivity than conventional imaging methods and can have a significant impact on management decisions, including surgical treatment, treatment intent, and local targeted treatment regimen [4,15,16], impacting more than 20% of patients with high negative predictive value (87%) [17]. The size of the nodal metastases can have an effect on the level of metabolic activity in affected nodes, reducing the uptake threshold for smaller nodes may be necessary, and rigid standardized uptake value (SUV) cut-offs may result in false-negative interpretations [18]. The location of the primary tumor may increase the risk of nodal metastases in certain levels of the neck, for example, level I in oral cavity cancers, level II in cancers of the pharynx, level VI in laryngeal cancers, and search patterns; while evaluating these imaging evaluations, one should be mindful of these findings [19]. Incorporating PET/CT in the pre-treatment evaluation of patients can lead to reduced rates of neck dissection, in comparison to conventional imaging or clinical findings-based approaches (7% vs. 44% vs. 90%) with associated significant impacts on treatment costs [20], and more importantly, similar outcomes in comparison to elective neck dissection [21]. The HPV status of the cancer can have an impact on the metabolic activity of the nodal disease, with HPV-negative tumors having comparatively lesser uptake, with different hypotheses stating increased cell proliferation, increased oxygen in the soft tissue milieu in HPV-positive tumors, and the increased incidence of cystic or necrotic nodes in HPV-negative tumors as potential causes [22].

## 5. Distant Disease Detection

**Figure 2:**
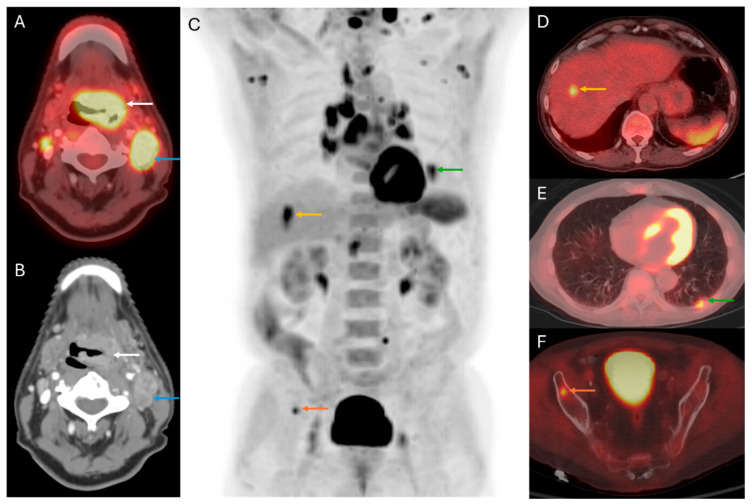
Distant disease detection and Prognosis: 74-year-old male with base of tongue SCC. Axial fused PET/CT (**A**,**D**–**F**), axial CT (**B**), and coronal MIP (**C**) images of the staging 18F-FDG PET/CT demonstrate the primary tumor in the tongue base (white arrows), cervical nodal metastases (blue arrows), hepatic (yellow arrows), pulmonary (green arrows), and osseous (orange arrows) metastases. Despite systemic therapy, the patient succumbed to the disease approximately 1.5 years after the examination.

The most significant contribution to patient management and outcome in head and neck cancer from ^18^F-FDG PET is probably the detection of distant disease, which is often not detected on conventional imaging and clinical examination. A meta-analysis reported high sensitivity (89%) and specificity (94%) for PET/CT in the detection of distant disease in patients with head and neck cancer in 1276 patients, where ^18^F-FDG PET/CT detected distant disease or a second primary malignancy in approximately 14% patients [23]. Similar diagnostic performance was reported in other studies for both PET/CT and PET/MRI, although the incidence of distant disease has been reported to be less frequent at staging than in previously treated patients [24,25]. Detection rates for distant disease are higher in advanced-stage tumors (stage III or IV) in comparison to early-stage disease (16% vs. 10%). PET/CT also detected more distant disease than chest X-ray combined with head and neck MRI, or chest CT combined with head and neck MRI [26]. The higher rates of PET/CT can be explained by the detection of metastases outside the thorax, with studies reporting similar performance for detecting disease within the chest [27]. In recent years, PET/MR has gained interest in the evaluation of head and neck cancers, although PET/MR may provide superior evaluation of the soft tissue extent and characteristics of the primary tumor, the detection rates of distant disease appear comparable between PET/MR and PET/CT [28]. Pulmonary, followed by osseous and distant nodal, metastases appear to be the most detected distant disease [29] (Figure 2).

## 6. Occult Primary and Synchronous Primary Malignancy Detection

PET/CT can detect the primary tumor in approximately 40–50% of patients with a clinically undetected primary squamous cell carcinoma of the head and neck [30,31], with high sensitivity (94%) and somewhat lower specificity (65%) [32]. Specificity can be improved with the addition of diffusion weighted imaging (DWI) and dynamic contrast enhancement (DCE) MR imaging [33]. The most detected occult primary location appears to be the oropharynx, involving the tongue base and the pharyngeal tonsils [34]. Normal physiological tonsillar metabolic activity shows no significant difference. However, in patients with an occult primary tumor, the affected tonsil demonstrated asymmetrically increased uptake in comparison to the normal contralateral side. A study reported a difference of 0.9 to 11.1 SUVmax in comparison to the normal contralateral side, while the difference in the physiologic normal uptake was 0.01 to 2.7 SUVmax, with a cut-off of 0.8 resulting in 100% sensitivity and 81% specificity, while a cut-off of 2.7 yielded 80% sensitivity and 100% specificity [35].

**Figure 3:**
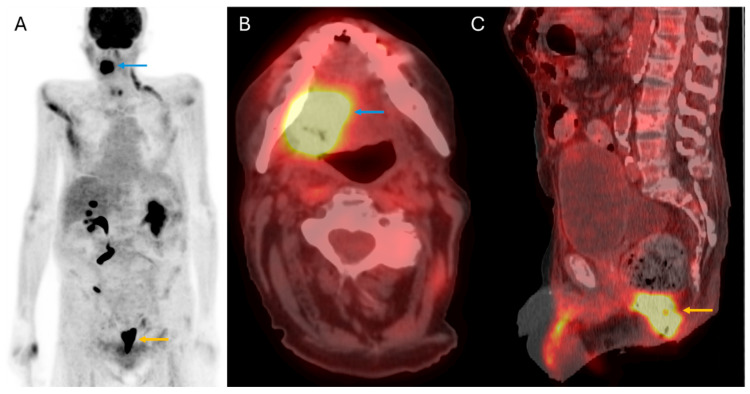
Synchronous primary malignancy detection: 70-year-old male with recurrent SCC of the oral cavity. Coronal MIP (**A**), axial fused PET/CT (**B**), and sagittal fused PET/CT (**C**) images demonstrate the primary recurrent malignancy in the oral cavity (blue arrows) and an incidentally detected hypermetabolic mass in the anal canal (yellow arrows), with a biopsy showing anal squamous cell carcinoma.

Although the incidence rates are low, PET/CT can detect a second primary malignancy in about 7–10% of patients with high sensitivity (100%), specificity (98%), and diagnostic accuracy (98%), with head and neck, lung, and aerodigestive tract cancers being the most detected synchronous primary tumors. However, the diagnostic performance can vary depending on the location of the second primary malignancy. Cancers of the oral cavity and hypopharynx have been shown to have a higher association with a synchronous second primary malignancy [36,37,38]. The performance decreases in esophageal malignancies and is limited by the early stage of these tumors, and endoscopic evaluation may be necessary to detect synchronous primary tumors in these regions [39] (Figure 3).

## 7. Role of PET/CT in Treatment Planning

PET/CT findings can result in a change in management plan in up to one-third to half of the patients, with the most impact on nodal staging, followed by primary tumor staging and distant metastasis detection. This impact is higher in patients with advanced nodal disease (N3) and, as expected, patients with unknown primary tumors [8,40]. Patients with a change in staging and subsequent treatment plan from the PET/CT findings had significantly worse outcomes [41]. PET/CT can help tailor surgical management in patients who are candidates for primary surgical treatment of the neck. In patients with a clinical N0 neck at diagnosis, PET/CT findings have a high negative predictive value (94%) and can result in a change in surgical plan in 21.5% patients, modifying the surgical plan with additional surgical neck dissection (12%) or reduced extent of neck dissection (5%) [17,19]. In radiation treatment planning, PET/CT has a significant impact on the treatment volume delineation, both for the primary tumor and nodal disease, in comparison to CT-based treatment volumes [42]. ^18^F-FDG PET/CT can predict the tumor response, identify patients at risk of treatment failure prior to radiation or chemoradiation therapy, and help tailor treatment planning [43,44]. PET/CT-based radiation treatment planning improved outcomes with better local tumor control in comparison to CT-based tumor volumes [45,46]. PET/CT-based gross tumor volumes can identify tumors outside CT-based tumor volumes, thereby improving the accuracy of radiation treatment planning [47]. The use of PET/CT-defined tumor volumes can be susceptible to partial volume effects, especially in smaller tumors, and this should be taken into account during radiation treatment planning [48]. PET/CT can provide functional characteristics of the tumor that can be used in intensity-modulated radiation therapy (IMRT) to target more metabolically active tumor subsites, which can decrease in-field recurrent malignancy [49]. In the recent past, non-FDG PET radiotracers that characterize tissue hypoxia have been used to help target radioresistant hypoxic tumor tissues [50].

## 8. Treatment Response Assessment and Recurrent Disease Evaluation

Treatment response assessment plays an important role in the management algorithm of head and neck cancer patients following primary treatment, either surgical, radiation, systemic therapy, or a combination of these. A meta-analysis including 1293 patients with HNSCC showed a sensitivity, specificity, and odds ratio of 85%, 93%, and 76, respectively, with lower sensitivity and specificity in HPV positive tumors, which may be related to the slower regrowth of treatment-resistant tumors and a higher incidence of immune-related inflammatory ^18^F-FDG uptake [51]. PET/CT has a high negative predictive value, with a negative post-treatment PET indicating a complete treatment response [52]. ^18^F-FDG PET/CT for treatment response evaluation is ideally performed about 3 months after completion of treatment to decrease the chances of false-positive interpretation secondary to post-treatment inflammation [53]. However, recent studies have shown mid-treatment (week 3 of radiation therapy) changes in DWI and PET/CT metabolic tumor volume (MTV) to be predictive of local recurrence after completion of treatment, with recurrence rates up to 78% in patients with unfavorable response on these imaging findings [54]. Similarly, early PET/CT after induction systemic therapy can help decide the treatment strategy after induction therapy by evaluating changes in metabolic tumor parameters [55]. ^18^F-FDG PET/CT can detect residual or recurrent diseases after definitive chemoradiotherapy with higher sensitivity than DWI-MR (100% vs. 90%), while DWI can provide higher specificity. A combination of these modalities can detect treatment response assessment with a high diagnostic accuracy [56]. In recent years, circulating tumor DNA (ctDNA) has been used in combination with PET/CT to evaluate for residual or recurrent disease, with a lower false-positive rate with ctDNA, and ctDNA kinetics during treatment can predict response [57,58].

**Figure 4:**
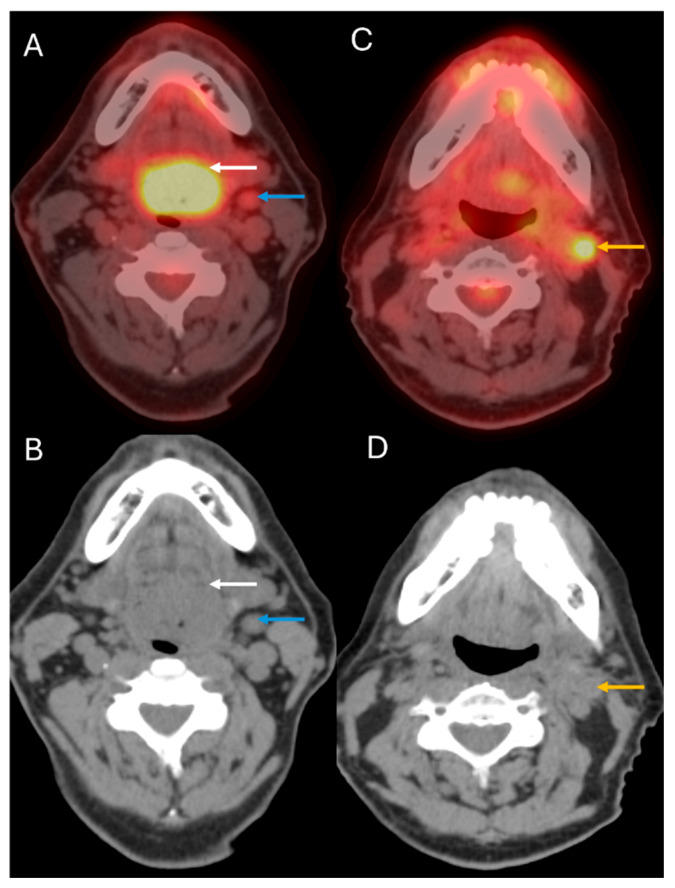
Recurrent disease detection: 63-year-old man with APV-negative SCC of the oropharynx treated with chemoradiation. Axial fused PET/CT (**A**) and axial CT (**B**) of the staging 18F-FDG PET/CT demonstrate the primary tumor (white arrows) and left cervical nodal metastasis (blue arrows). Axial fused PET/CT (**C**) and axial CT (**D**) images of the restaging PET/CT performed approximately 5 years after treatment completion for a palpable neck node demonstrate recurrent left cervical nodal disease (yellow arrows).

Over the years, many response assessment criteria using quantitative, semi-quantitative, or qualitative methods to assess treatment response have been proposed. Some of these are the Positron Emission Tomography Response Criteria in Solid Tumors (PERCIST); Neck Imaging Reporting and Data System (NI-RADS), which uses qualitative assessments of the uptake in the primary tumor and lymph nodes; the Hopkins criteria, a qualitative 5-point scale that uses blood pool activity and liver activity as reference regions to classify lesion uptake, with lesions showing focal uptakes greater than the liver being suspicious for residual malignancy; and changes in quantitative PET measurements, such as ΔSUVmax, ΔMTV, and ΔTumor Lesion Glycolysis (TLG). These have all been shown to predict patient outcomes at the treatment response assessment [59,60]. These criteria have been evaluated with multiple validation studies showing a high diagnostic accuracy in detecting residual/recurrent disease after primary treatment [61]. With the increasing use of immunotherapeutic agents in the treatment of head and neck cancers, it should be kept in mind that false-positive nodal uptakes can be seen and should be considered [62,63] (Figure 4).

## 9. Role of PET/CT in Prediction of Prognosis

Being able to predict patient outcomes can provide valuable information that can be used to tailor treatment and identify patients at risk of recurrent disease and patients who may need additional or more frequent follow-up after treatment completion. A meta-analysis of 26 studies evaluating the prognostic value of PET/CT performed during or after treatment showed a strong correlation between PET/CT findings and mortality or progressive disease at the 2-year mark and up to 5 years after completion of treatment. A 2-year mortality risk ratio of 8.3 for the post-treatment PET/CT and 4.0 for intratherapy PET/CT was noted [64]. PET/CT has a prognostic predictive value at baseline prior to treatment as well as post-treatment, with lower SUVs being associated with better disease-free survival, overall survival, and local control [65]. High nodal 18F-FDG uptake (nodal SUVmax above 8.7 g/mL) at baseline has been shown to be associated with the presence of distant metastatic disease [66]. High 18F-FDG uptake (SUVmax ≥ 2.7; HR 7.0 for local control and 9.7 for cause-specific survival) after treatment is associated with poor survival and local disease control [67]. In patients with recurrent HNSCC, PET/CT is able to predict worsening prognosis in patients with localized, locally advanced, or metastatic disease, whereas conventional imaging methods are unable to do so, with PET/CT showing a superior predictive ability for cancer-specific mortality [68]. PET/CT outperforms conventional imaging methods in predicting tumor control after treatment, probably secondary to the valuable functional information provided by PET [69,70]. PET/CT response assessment during induction chemotherapy can help differentiate responders from non-responders who may need more intense treatment strategies and provides important survival information [71,72].

## 10. PET/CT in the Follow-Up of Head and Neck Cancer

Follow-up imaging recommendations are dependent on tumor aggressiveness, staging at diagnosis, prognostic information, etc. The value of PET/CT when the post-treatment PET assessment is negative in an asymptomatic patient with a negative clinical exam has not been established [6]. PET/CT has high sensitivity and specificity in detecting recurrent disease during the follow-up of HNSCC [73]. Abgral et al. showed a sensitivity (100%), specificity (85%), positive predictive value (77%), negative predictive value (100%), and diagnostic accuracy (90%) for follow-up PET/CT in the detection of recurrent disease performed approximately 12 ± 4 months after treatment completion [74]. At a mean follow-up of 11 months after the completion of treatment, Li et al. reported a diagnostic accuracy of 88%, in comparison to 66% for conventional imaging techniques [75]. Similar findings were reported by others [76].

## 11. Conclusions

The role of ^18^F-FDG PET/CT in the evaluation of patients with head and neck squamous cell carcinomas has been well established and is constantly emerging. It has an added advantage over conventional imaging techniques in many situations, including the detection of synchronous and occult primary tumors, and facilitates accurate staging with the detection of nodal and distant metastatic disease. PET/CT also plays a role in treatment planning, including surgical, radiation, and systemic treatment planning. It has proven to be a valuable tool in the follow-up of these patients by detecting recurrent and metastatic disease. Prognostic information can be derived from PET/CT findings, which, in turn, can have a significant impact on patient management and treatment strategies.
